# Supplementary material for: The Role of Cocrystallization-Mediated Altered Crystallographic Properties on the Tabletability of Rivaroxaban and Malonic Acid
Source: Pharmaceutics. 2020 Jun 12;12(6):546. doi: 10.3390/pharmaceutics12060546 (PMC7356764; doi:10.3390/pharmaceutics12060546)
Supplement: Supplementary file 1 [file pharmaceutics-12-00546-s001.pdf]

pharmaceutics

Supplementary Materials: The Role of Cocrystallization-Mediated Altered Crystallographic Properties on the Tabletability of Rivaroxaban and Malonic Acid

Dnyaneshwar P. Kale, Vibha Puri, Amit Kumar, Navin Kumar and Arvind K. Bansal

1. Identification of Slip Planes

This has been thoroughly discussed in the main manuscript. It is necessary to mention that the prediction of (0 1 1) as primary slip plane using "Dreiding with current charge" for RIV is consistent with the visualization method (Table S1).

Table S1. Slip plane identification by different methods.

Compound	CCDC Code	Primary Slip Planes Identification by						
		Visualization	COMPASS		Dreiding Qeq		Dreiding Current Charge	
			(h k l)	E_{att} in kcal/mol	(h k l)	E_{att} in kcal/mol	(h k l)	E_{att} in kcal/mol
RIV-MAL Co	1854618	(0 0 1)	(0 0 1)	−28.4	(0 0 1)	−33.8	(0 0 1)	−17.8
RIV	1854617	(0 1 1)	(0 0 1)	−39.9	(0 0 1)	−44.1	(0 1 1)	−26.9
-	-	-	(0 1 1)	−44.2	(0 1 1)	−55.8	(0 0 1)	−30.1
MAL	1209218	absent	(0 0 1)	−28.4	(0 0 1)	−33.8	unstable surfaces[a]	

[a] The calculations could not succeed and get terminated due to unstable surfaces.

2. Heckel Analysis

A linear portion of Heckel plot was used for calculating the "mean yield pressure" of the three solids and the fitted lines to the linear regions are shown in following curves.

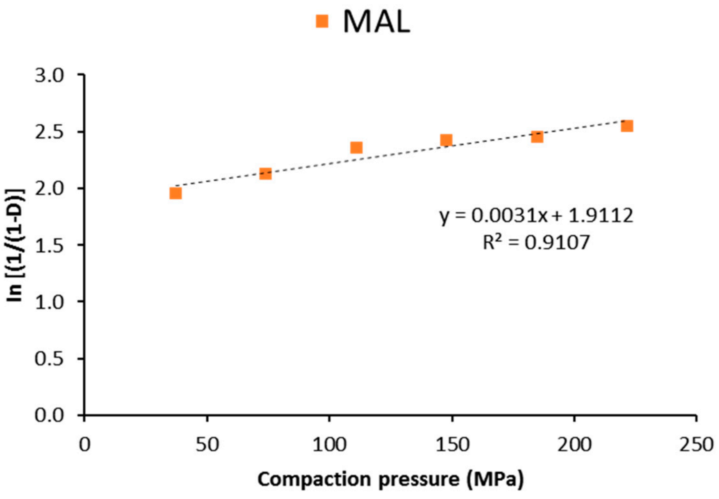

Figure S1. Heckel plot fitted for estimating Py value of for MAL.

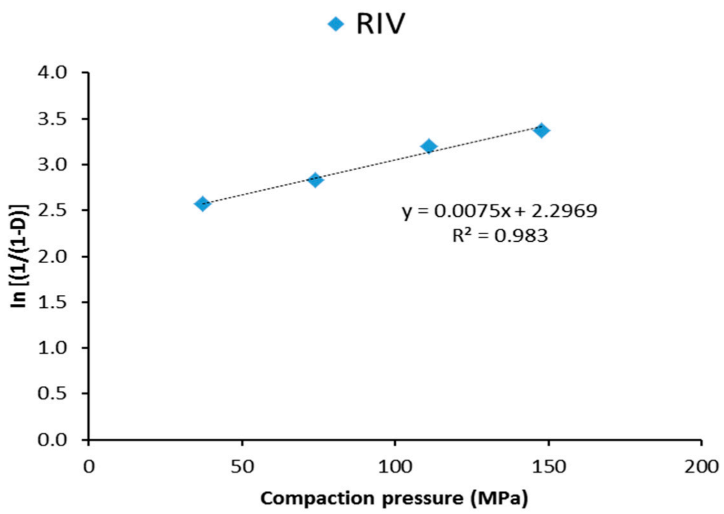

Figure S2. Heckel plot fitted for estimating Py value of RIV.

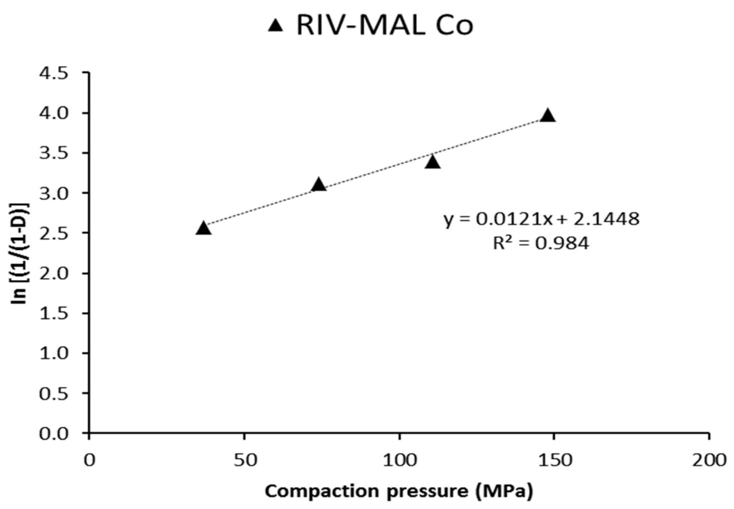

Figure S3. Heckel plot fitted for estimating Py value of RIV-MAL Co.
